# Harnessing the Immunomodulatory Effects of Radiation in Urinary Bladder Cancer

**DOI:** 10.7759/cureus.4108

**Published:** 2019-02-20

**Authors:** Waseem Abbas, Vineeta Goel, Arun Verma, Vineet G Gupta, Ranga R Rao

**Affiliations:** 1 Medical Oncology, Max Institute of Cancer Care, New Delhi, IND; 2 Radiation Oncology, Max Institute of Cancer Care, New Delhi, IND

**Keywords:** abscopal effect of radiation and immunotherapy, immunomodulatory effect of radiation and check point inhibtors in urinary bladder

## Abstract

The use of local radiation to elicit distant tumor response was proposed long back. The abscopal effect is regression of non-irradiated metastatic lesions at the distant site and there is an enormous therapeutic effect of immunomodulation. Radiation causes cancer cells to release antigens which mount an immune response, but this response is short lasting because cancer cells evade recognition by different mechanisms. Programmed Death Ligand-1 (PDL-1) pathway has been extensively studied. Combining immunotherapy and radiotherapy may result in long-term remissions especially for stage 4 cancer. Here we present a case of high grade urothelial carcinoma that progressed after four cycles of chemotherapy and after giving palliative radiation to urinary bladder he was started on nivolumab. First scan done after radiation and six cycles of nivolumab showed complete response. The patient continues to be in remission for the last 17 months from the start of radiation and immunotherapy that was started sequentially. Overall survival till date is 25 months.

## Introduction

With the advent of immunotherapy, there is a lot of ongoing debate as to how radiation induces cell death. Radiation to elicit an immune response for abscopal effect will be beneficial for treatment. There are different forms of cell death which we know as necrosis, necroptosis and autophagic cell death which have been extensively studied and this process activates innate immunity which lays the foundation for cascade of immunological reactions [1-3]. How do cells die after radiation? Radiation causes mitotic catastrophe along with cellular senescence which results in loss of only proliferative capacity. Whereas we know true cell death means that there will be loss of both morphological and genomic integrity and that elicits the immunological response. What will induce the immunogenic response that we need? There are many ongoing studies pertaining to this topic but the ones most extensively studied are AIM2 (Absent In Melanoma 2) recognition of deoxyribonucleic acid (DNA) damage and cGAS (Cyclic GMP-AMP Synthase) recognition of DNA damage with activation of sling [4]. Many case reports talk about abscopal effects of radiation and it has been seen that it is immunologically mediated [5,6]. Postow et al. published a case report of a 33-year-old female [7], who was progressing on ipilimumab, was given radiation and this slowed the response in the radiation lesion and at other sites as well. Keynote -001 showed that patients who received radiotherapy before starting immunotherapy had better survival (10.7 vs 5.3). This means that tumor antigens are liberated after radiation, and T cells which are exhausted become more effective and mount better anti-tumoral response. At which radiation dose will this happen remains a big question and different strategies have been tried [7-9]. There are a very few studies with check point inhibitors and the dose of radiation which is yet to be properly investigated, and so far the dose performing well in abscopal effect is 8 Gy (Gray) in three fractions [4]. The strongest effects have been seen with hypo fractionated regimens. Other question is whether the site to be irradiated is liver, lung, bone, or lymph nodes and data stands more with the visceral organs [10]. Immune privilege is another factor on which the much desired abscopal effect depends. Bone marrow has been proposed as a site of immune privilege [11] where hypo fractionation may not work as compared to other organs. Can radiation before the administration of immunotherapy be detrimental in some cases? A lot of questions remain unanswered. But with more trials and case reports pointing to the benefits, questions such as the best method of sequencing, the dose required and the target to be irradiated will be answered in due time.

## Case presentation

**Figure 1:**
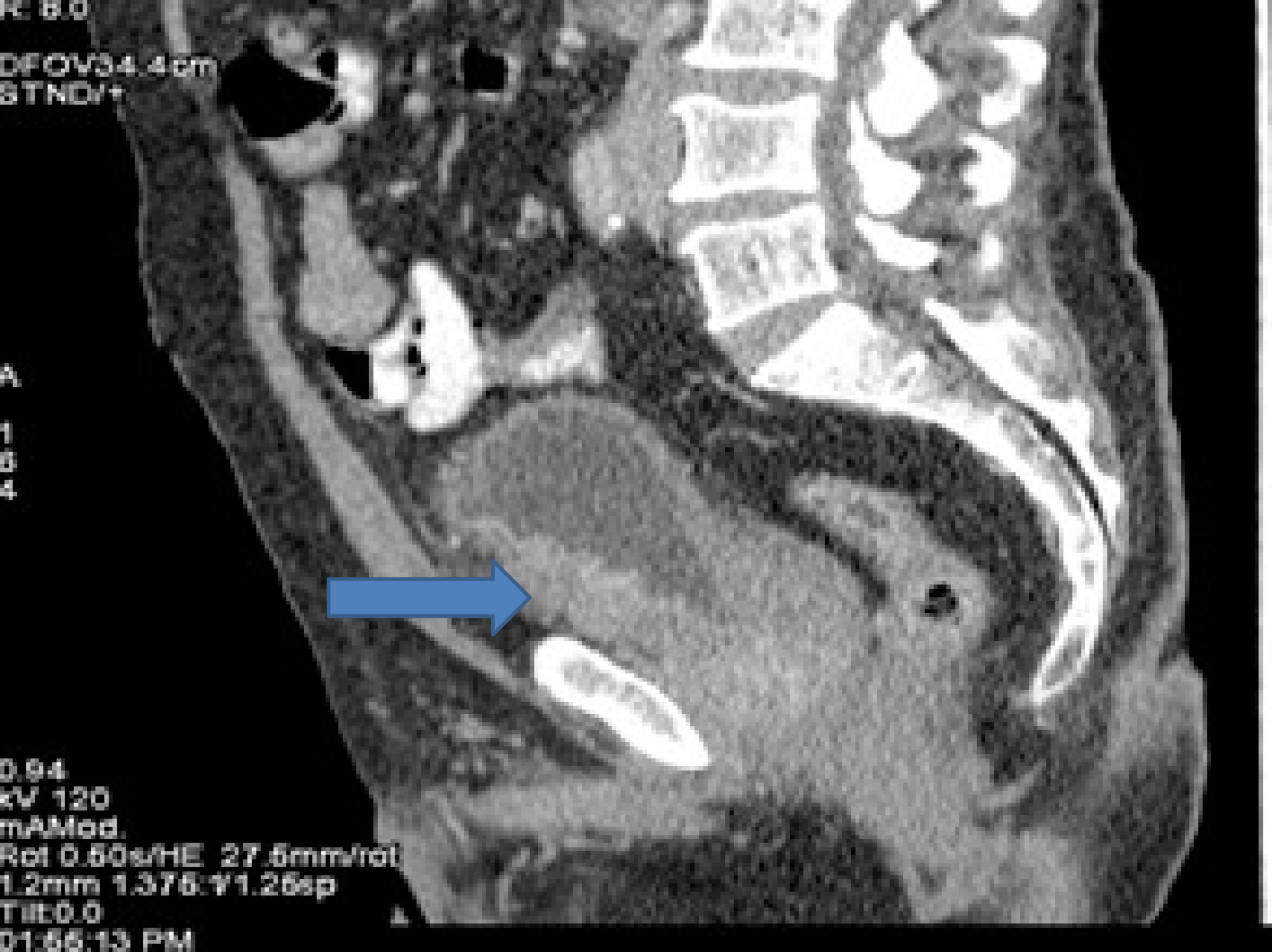
Computed tomography (CT) showing bladder cancer involving inferior half of urinary bladder with perivesical extension.

**Figure 2:**
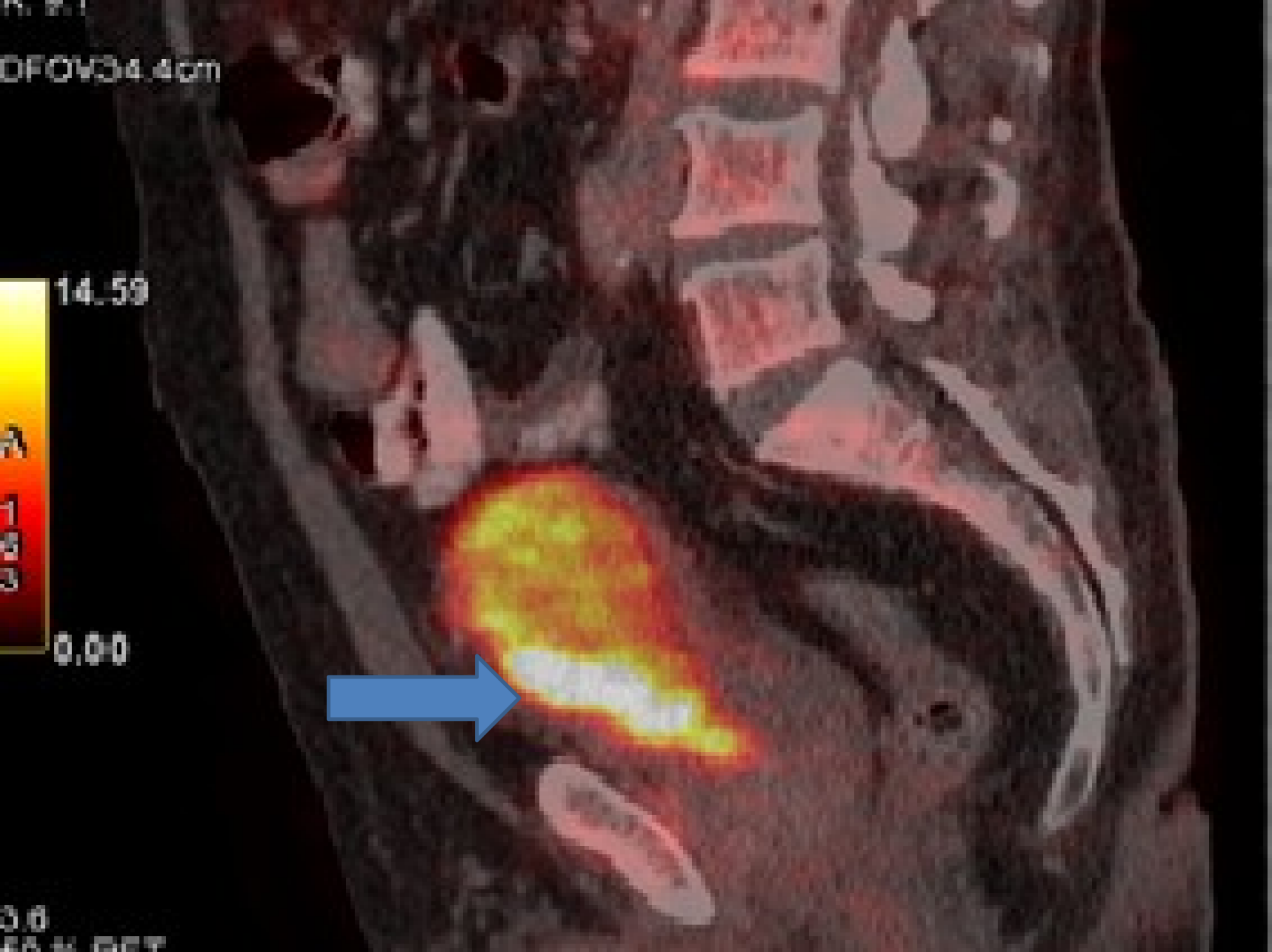
Positron emission tomography (PET) showing bladder cancer involving inferior half of urinary bladder with perivesical extension.

A 69-year-old male, non-diabetic, normotensive, smoker presented with hematuria in November 2016. Magnetic resonance imaging (MRI) pelvis showed intraluminal mass lesion involving the inferior half of urinary bladder, infiltrating its anterior wall, with perivesical extension along with sub-centrimetric pelvic lymphadenopathy. No metastatic disease outside pelvis was seen on positron emission tomography-computed tomography (PET-CT). Transurethral resection of bladder tumour (TURBT) could not be done because of extensive intravesical tumor growth and bleeding. Histopathology showed high-grade urothelial carcinoma. The patient was started on neoadjuvant chemotherapy with gemcitabine and carboplatin (Figures 1-2).

**Figure 3:**
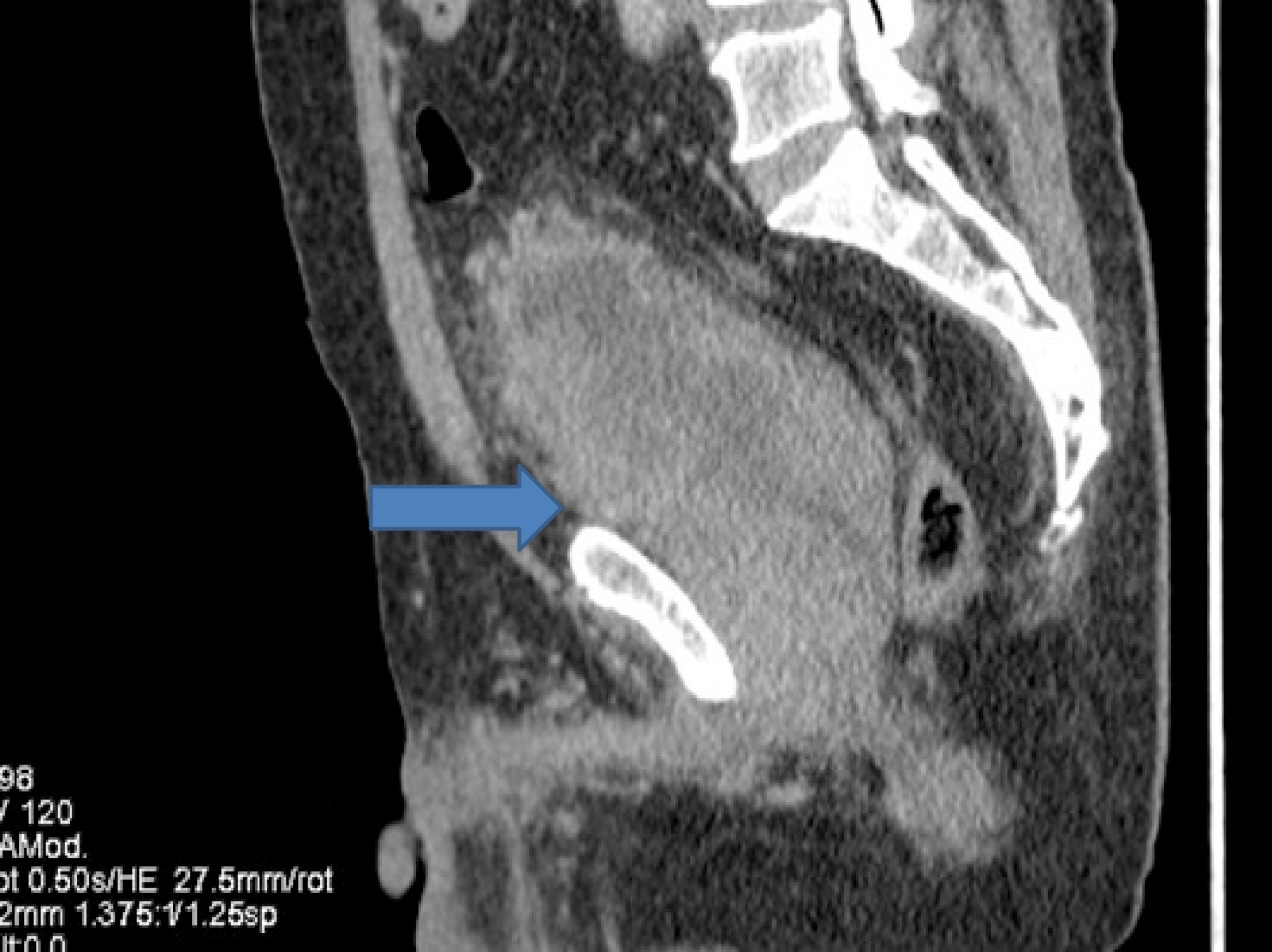
Computed tomography (CT) showing progression in urinary bladder.

**Figure 4:**
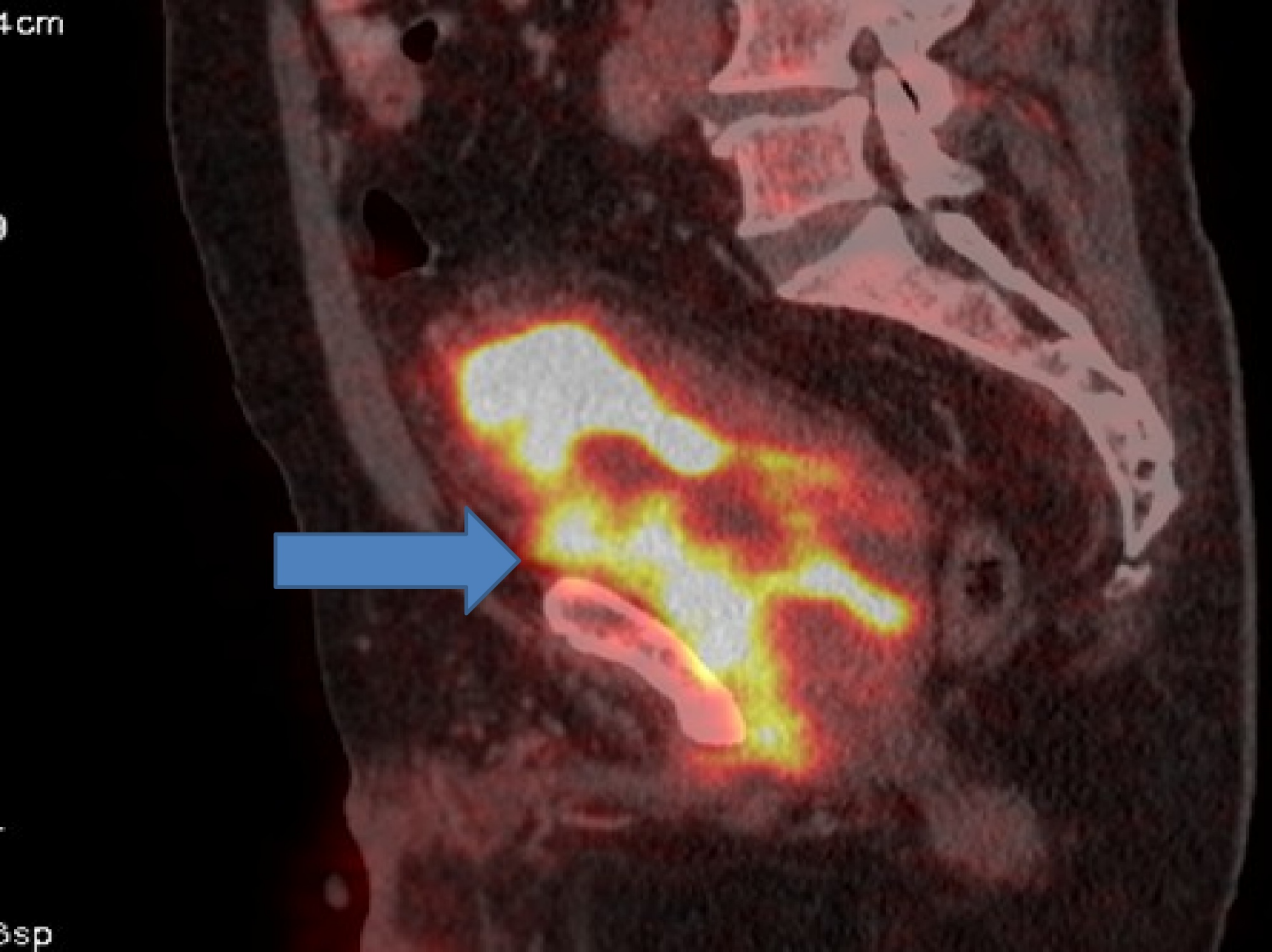
Positron emission tomography (PET) showing progression in urinary bladder.

**Figure 5:**
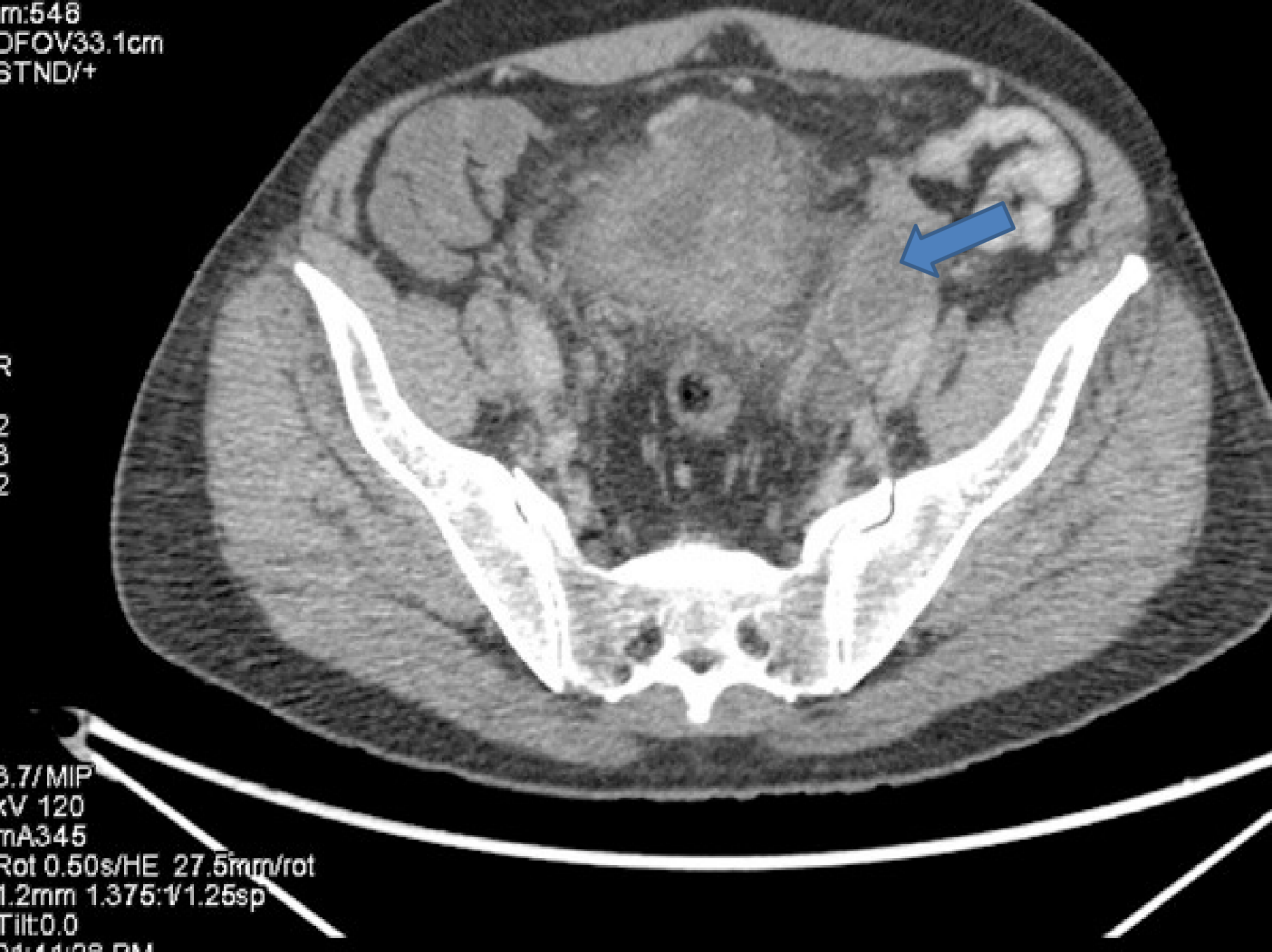
Computed tomography (CT) showing progression in left internal iliac lymph nodes.

**Figure 6:**
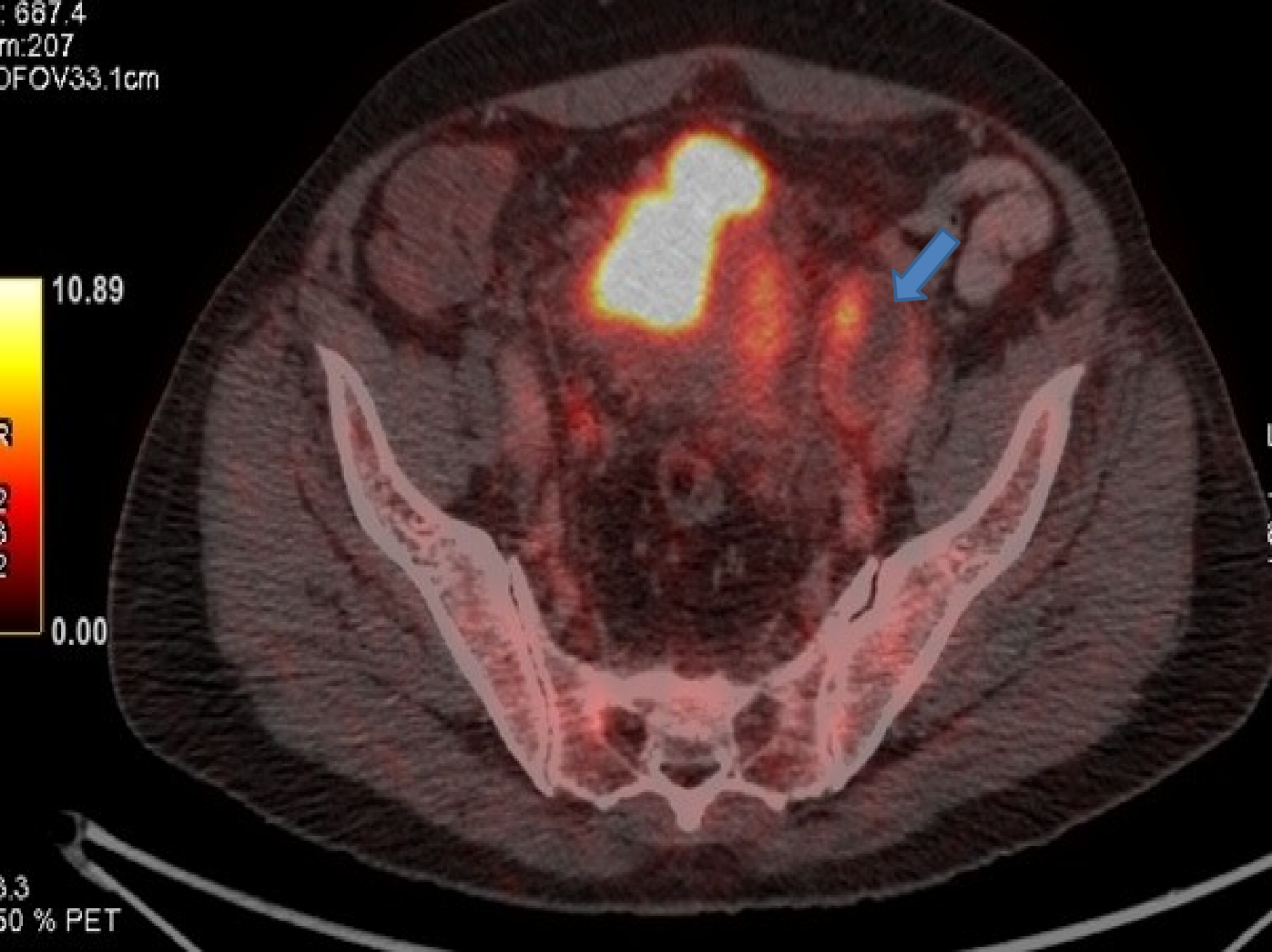
Positron emission tomography (PET) showing progression in left internal iliac lymph nodes.

**Figure 7:**
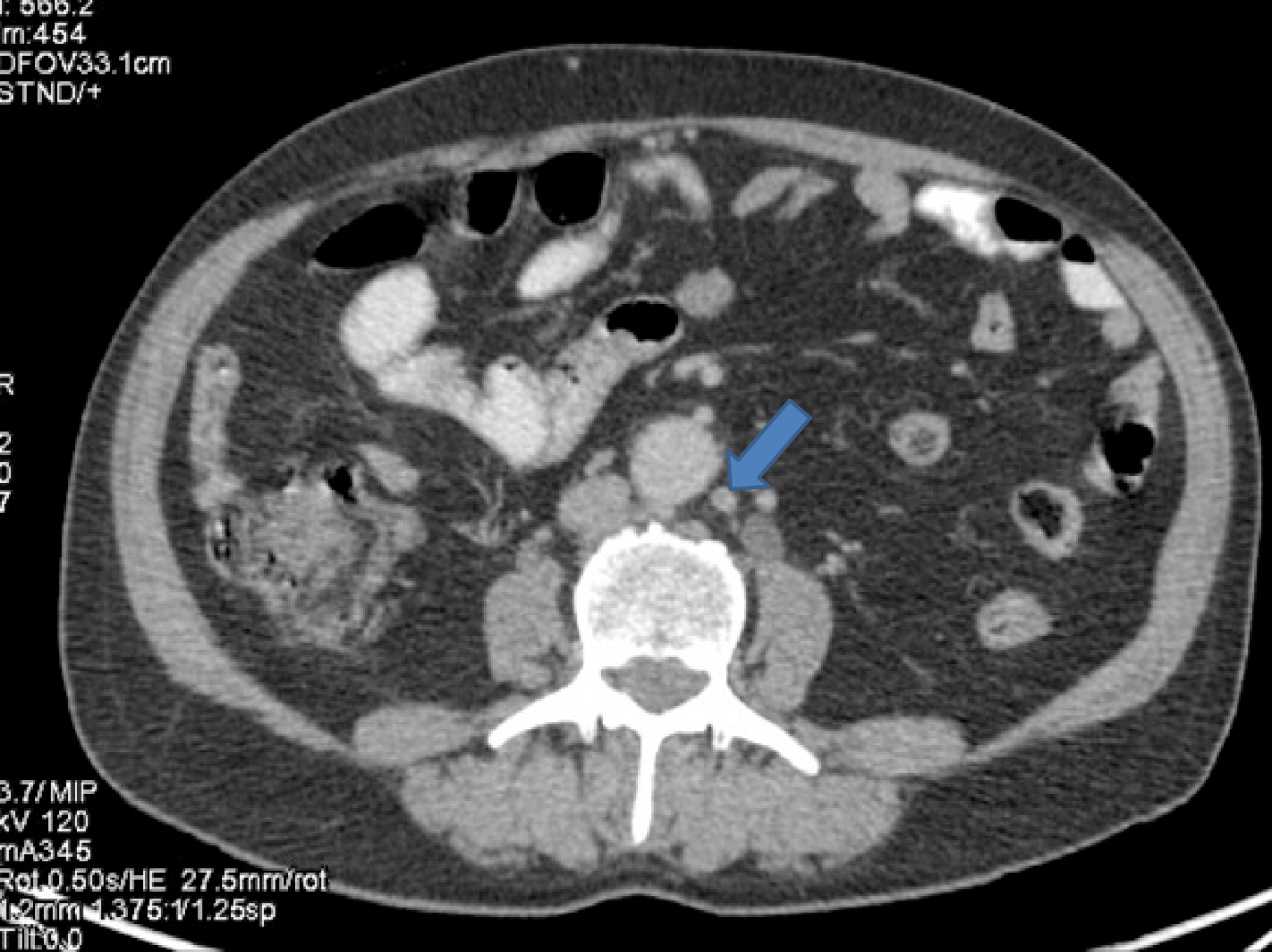
Computed tomography (CT) showing a para-aortic lymph node measuring about 1 cm from where fine needle aspiration cytology (FNAC) was positive for malignant cytology.

**Figure 8:**
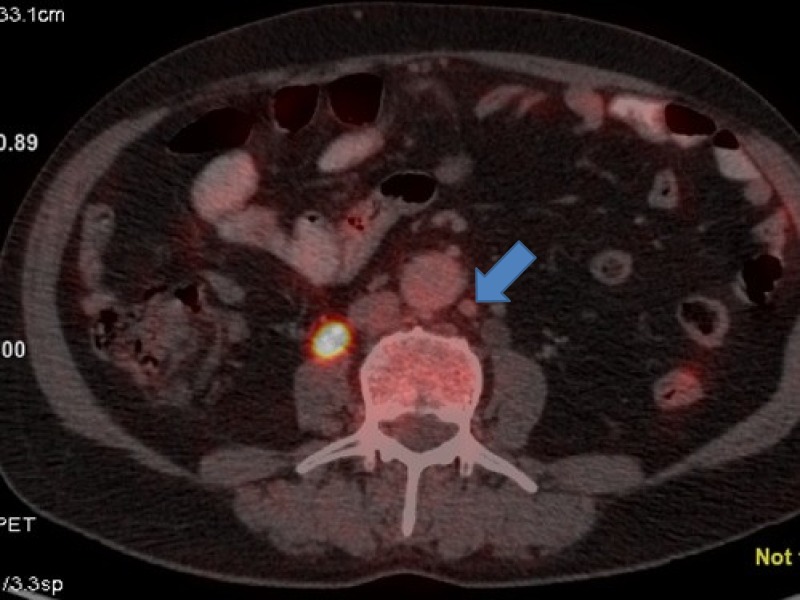
Positron emission tomography (PET) showing a para-aortic lymph node measuring about 1 cm from where fine needle aspiration cytology (FNAC) was positive for malignant cytology.

After four cycles, assessment revealed disease progression. PET-CT showed progression in urinary bladder with increase in extent of disease. Apart from urinary bladder, there was progression in left internal iliac lymph nodes largest measuring 3 cm. Internal iliac lymph nodes were increased both in size and fluoro deoxy glucose (FDG) avidity. Single para aortic lymph node measuring 1 cm was also a new finding. Fine needle aspiration cytology (FNAC) done was positive for carcinoma (Figures 3-8).

**Figure 9:**
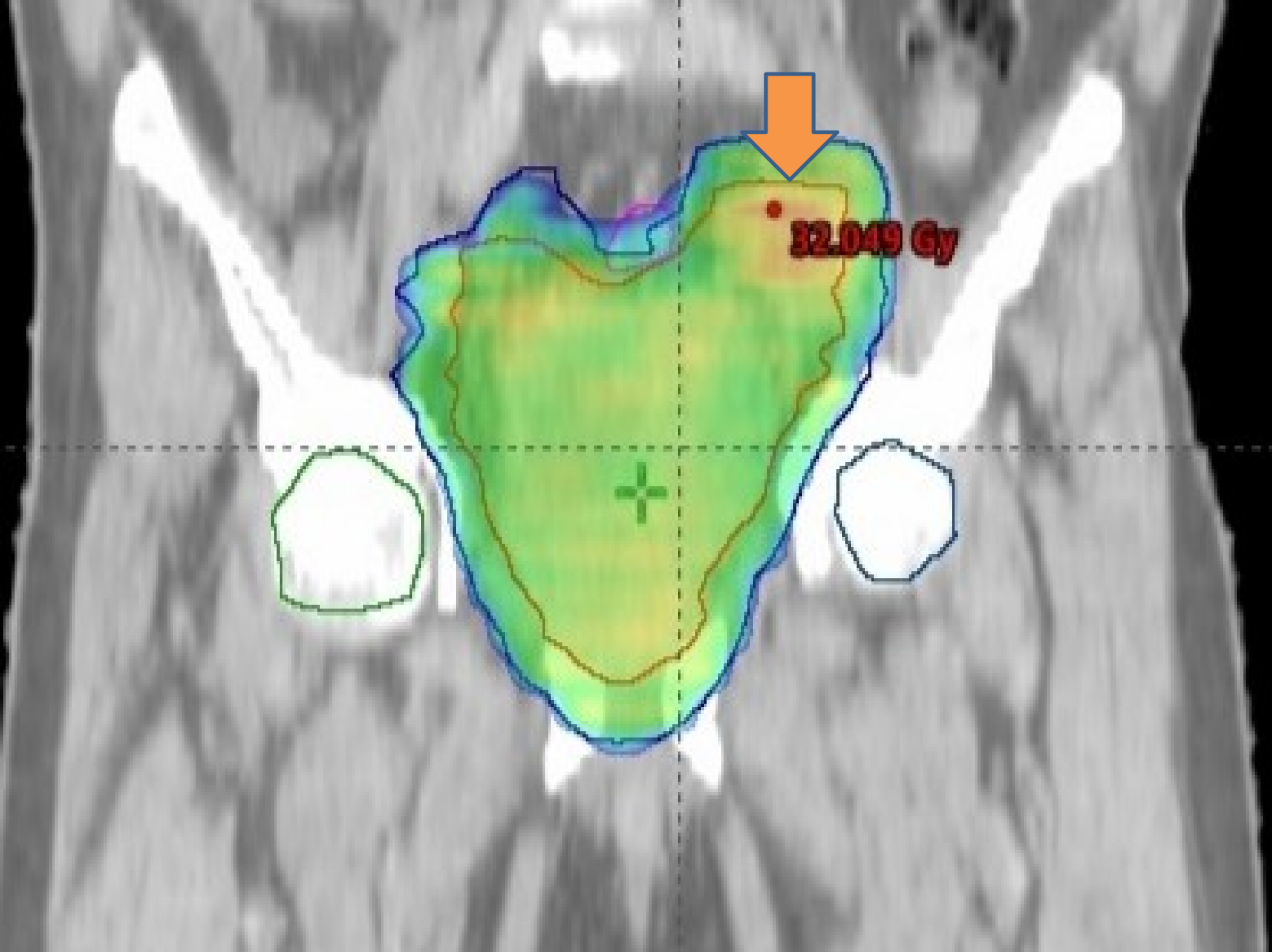
The figure showing radiation planning. Palliative radiation was given to bladder and internal iliac lymph node.

The patient developed severe pain in pelvic area, hematuria and recurrent urinary tract infection which deteriorated his performance status. The patient was started on palliative radiation to urinary bladder by image guided radiation therapy (IGRT) technique at the dose of 30 Gray (Gy) to urinary bladder and 32 Gy to left iliac lymph node in 12 fractions (Figure 9). Programmed Death Ligand 1 (PDL-1) was negative.

**Figure 10:**
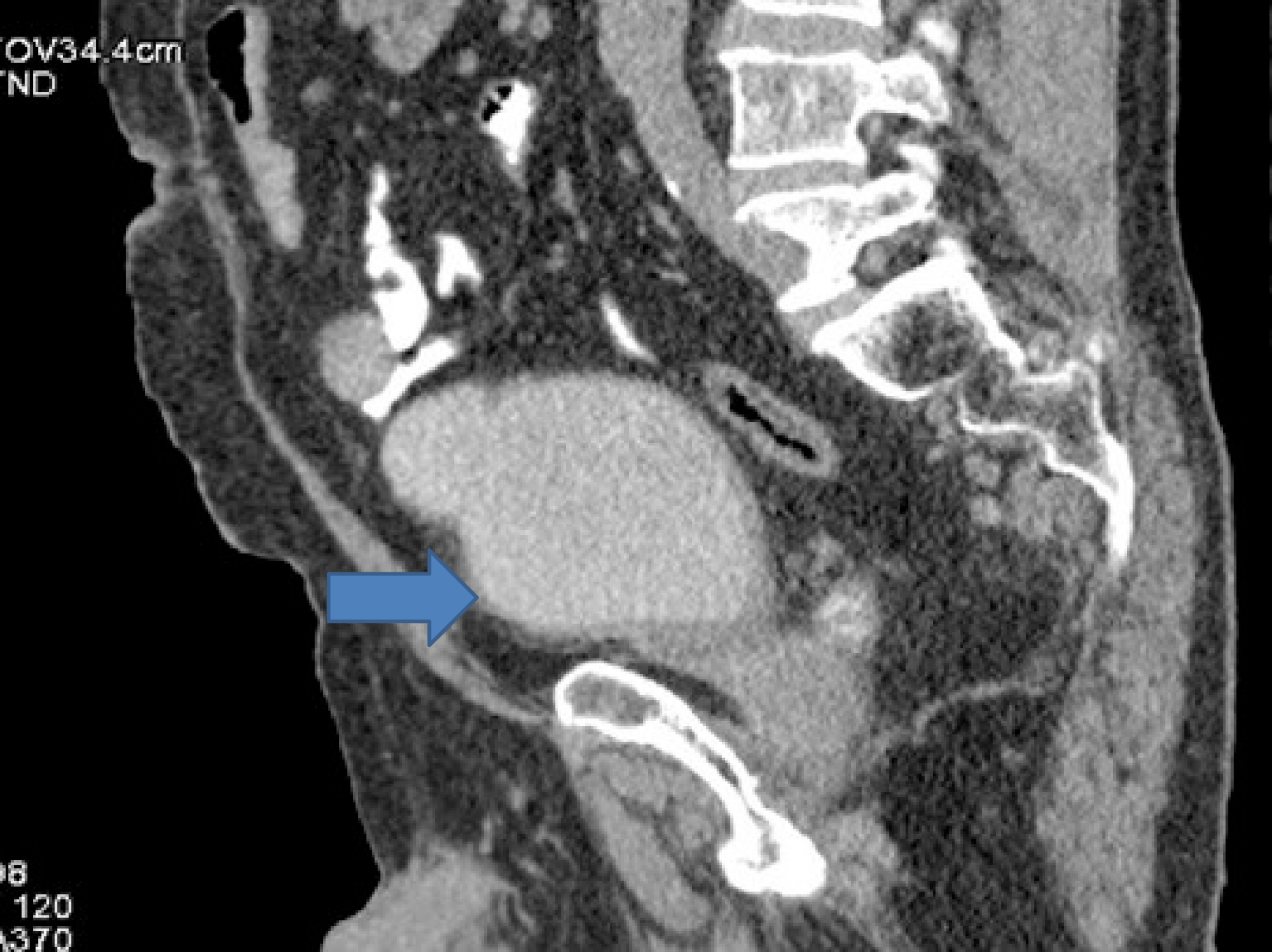
Computed tomography (CT) done after 17 months of starting radiation and immunotherapy showing complete remission in urinary bladder.

**Figure 11:**
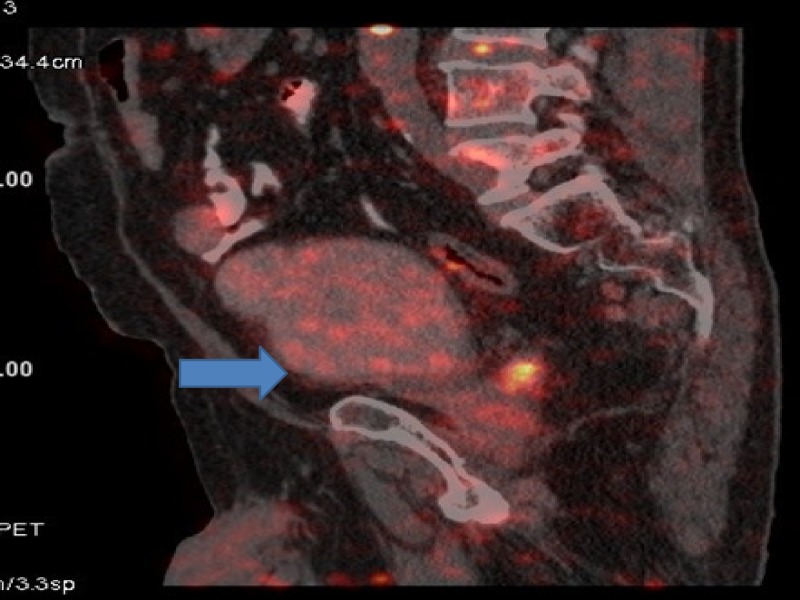
Positron emission tomography (PET) done after 17 months of starting radiation and immunotherapy showing complete remission in urinary bladder.

**Figure 12:**
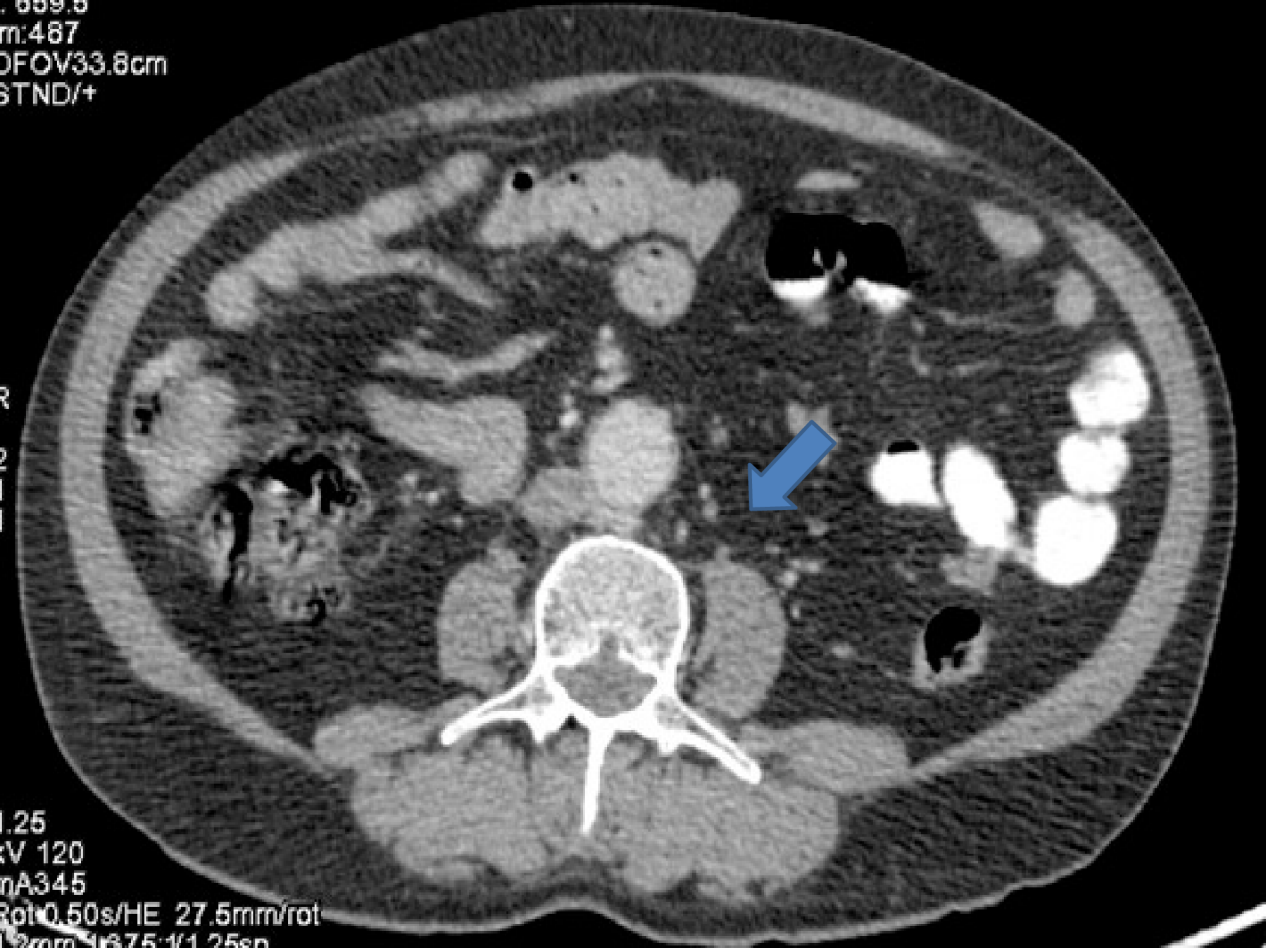
Computed tomography (CT) done after 17 months of starting radiation and immunotherapy showing disease in complete remission and no para-aortic lymph node was seen.

**Figure 13:**
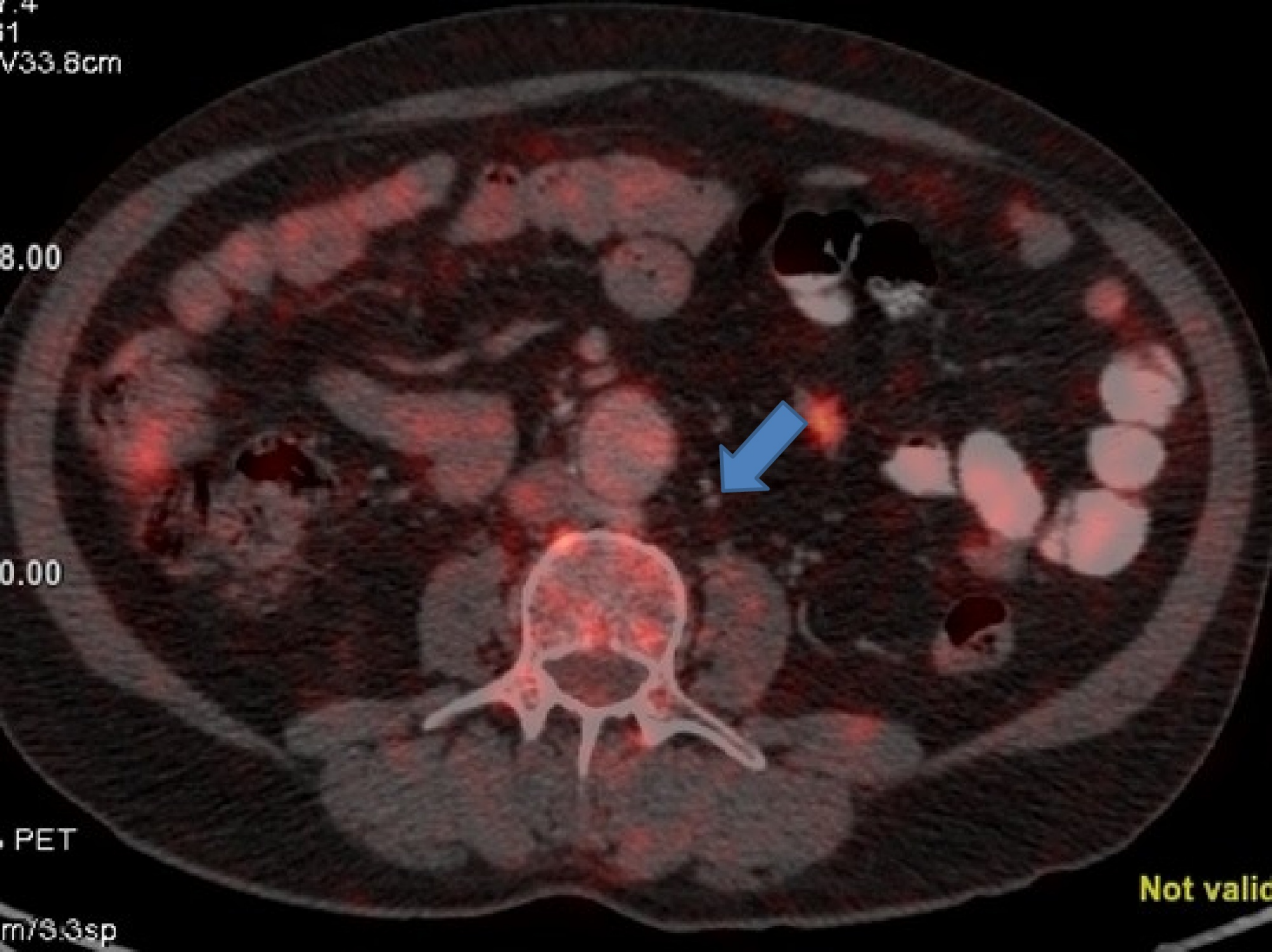
Positron emission tomography (PET) done after 17 months of starting radiation and immunotherapy showing disease in complete remission and no para-aortic lymph node was seen.

After completion of radiation, the patient was started on immunotherapy with nivolumab from August 2017. PET-CT done in December 2017 showed the disease was in complete remission (CR). The patient continues to be on nivolumab with no adverse events. Last assessment done in December 2018 showed that patient is in CR (Figures 10-13). Total progression-free survival (PFS) till December 2018 was 17 months. Overall survival till date is 25 months from the date of diagnosis.

## Discussion

Median survival for metastatic urinary bladder carcinoma is approximately one year with chemotherapy, less than six months without treatment and long-term survival is rare. No new therapy has been approved in over 30 years until the recent approval of immunotherapy. Still survival with immunotherapy remains poor. In a study by Bellmunt et al., median survival with pembrolizumab was 10.3 months in second line setting [12].

A different era of treatment for urinary bladder cancer has just begun. Focus is on immunomodulation. Immunotherapeutic agents are already approved for carcinoma urinary bladder. Although newer drugs are in pipeline to consolidate initial gains, the prognosis still remains poor for stage 4 urinary bladder and response rates are not great. If response does happen it is short lived. After disease progression not many options are available. So it is very important to control the disease at the start of treatment.

69-year-old male patient diagnosed with high grade urothelial carcinoma urinary bladder and subcentrimetric lymph nodes on PET-CT, progressing in and outside pelvis (para-aortic lymph node), after four cycles of platinum doublet presented to us with a very poor performance status (Eastern Cooperative Oncology Group - 3), severe pain in pelvic area, recurrent urinary tract infection and hematuria. The patient was given palliative radiation (30 Gy) to urinary bladder and internal iliac lymph node. After completion of radiation, he was started on immunotherapy with nivolumab. First scan was done after six doses of nivolumab and the patient was in complete remission in both pelvis and extra pelvis. Para-aortic lymph node was not seen on scan. The patient continues to be on treatment with nivolumab, and is in complete remission till date with last scan done in December 2018, 17 months from the start of radiation.

Few case series of abscopal have been published already but none for carcinoma urinary bladder. Still many questions are yet to be answered, like optimal dose and fractionation of radiation therapy (RT) in abscopal effects, combination, time window for RT and immunotherapy, biomarkers for predicting the abscopal effect. We need further more studies on combination of immunotherapy and radiation in carcinoma urinary bladder especially locally advanced.

## Conclusions

Extensive preclinical data supports the synergistic anti-tumor effect provided by immunotherapy and radiation therapy. In addition, recent case reports suggest that combining the two therapies can enhance the systemic anti-tumor effect. It is difficult for radiation therapy alone to overcome the immunoresistance of malignant tumors. Both the therapies combined double the anti-tumor effect which can be beneficial by increasing the overall survival (OS) as well PFS. Here we present a case of high-grade urothelial carcinoma that progressed after four cycles of chemotherapy and after giving palliative radiation to urinary bladder and the patient was started on nivolumab. First scan done after radiation and six cycles of nivolumab showed complete response. The patient continues to be in remission for last 17 months from the start of radiation and immunotherapy that was started sequentially. Many challenging questions are yet to be concluded regarding the optimal dose, optimal time and target to be irradiated. More evidence is required to combine immunotherapy and radiation therapy to be more clinically efficient which needs to be addressed in future clinical and preclinical trials.
